# Interactive effects of maternal exposure to chemical fertilizer and socio-economic status on the risk of low birth weight

**DOI:** 10.1186/s12889-022-13604-z

**Published:** 2022-06-16

**Authors:** Shiqi Lin, Jiajia Li, Jilei Wu, Fan Yang, Lijun Pei, Xuejun Shang

**Affiliations:** 1 Institute of Population Research/China Center on Population Health and Development, Peking University, Beijing, 100871 China; 2 Department of Andrology, School of Medicine, Jinling Hospital, Nanjing University, No.305, East Zhongshan Road, Nanjing, 210002 Jiangsu China

**Keywords:** Interactive effects, Maternal exposure to chemical fertilizer, Socio-economic status, Term low birth weight, Population-based case–control study

## Abstract

**Background:**

Maternal exposure to chemical fertilizer and disadvantaged maternal socio-economic status (SES) have been found to associate with increased risk of low birth weight (LBW). However, whether the two factors would interact to elevate the risk of LBW remains unknown. The present study aimed to explore the interactive effects of maternal exposure to chemical fertilizer during pregnancy and low SES on the risk of term LBW (tLBW).

**Methods:**

In this population-based case–control study, 179 tLBW cases (birthweight < 2500 g and gestational age ≥ 37 weeks) and 204 controls (birthweight ≥ 2500 g and gestational age ≥ 37 weeks) were chosen from the Perinatal Health Care Surveillance System of Pingding County, Shanxi Province, China between 2007 and 2012. Data on basic socio-demographic, dietary and lifestyle characteristics and environmental exposure were directly extracted from the system. Maternal exposure to chemical fertilizer was measured at both household level and village level. Household-level exposure was indicated by household chemical fertilizer use in farming during pregnancy and the data was collected by trained healthcare workers after the selection of cases and controls in 2013. Village-level exposure was indicated by annual amount of village chemical fertilizer consumption per acre and the data came from the Annals of National Economics Statistics of Pingding County in 2010. Interactions between maternal exposure to chemical fertilizer and SES were assessed in logistic regressions using relative excess risk due to interaction (RERI), which indicates an additive interaction if larger than 0.

**Results:**

The combination of low maternal SES and high exposure to village-level chemical fertilizer consumption was associated with increased risk of tLBW (aOR = 2.62, 95%CI: 1.44 ~ 4.77); The combination of low maternal SES and exposure to household chemical fertilizer use was associated with elevated risk of tLBW (aOR = 2.18, 95%CI: 1.24 ~ 3.83). Additive interactions were detected between high exposure to village-level chemical fertilizer consumption and low maternal SES (RERI:1.79, *P* < 0.001) and between exposure to household chemical fertilizer use and low maternal SES (RERI:0.77, *P* < 0.05).

**Conclusions:**

Our study suggested negative impacts of potential agricultural pollutants on adverse pregnancy outcomes, especially in disadvantaged socio-economic populations.

**Supplementary Information:**

The online version contains supplementary material available at 10.1186/s12889-022-13604-z.

## Background

Low birth weight (LBW), normally referring to babies weighing less than 2,500 g at birth [1], is one of the major public health concerns across the globe. Studies have shown that neonatal death among infants weighing 1,500 ~ 2,500 grams is 20 times higher than that among infants of normal weight (2,500 ~ 4,000 grams) [2]. Moreover, LBW has found to be associated with metabolic syndromes, cardiovascular diseases, respiratory diseases, and neural and cognitive disorders in later life [1]. It is estimated that around 15% of newborns were LBW globally [1] and the incidence of LBW in mainland China has been rising from 5.87 per 100 births in 2000 to 6.1 per 100 births in 2011 [3].

As one of the world’s largest users of chemical fertilizer during the past decade [4], China is now concerned about the potential health risk brought about by overuse of chemical fertilizer. As high as 68% of heavy metals in soil, including cadmium (Cd), mercury (Hg), arsenic (As), copper (Cu), lead (Pb), chromium (Cr), zinc (Zn) and nickel (Ni), can be attributed to application of chemical fertilizer, with phosphate fertilizer contributing most to the pollution [5]. It’s estimated that over 13% of grain production is affected by the heavy metal pollution in farmland soil [6]. Besides, application of chlorine-containing or nitrogenous fertilizer could change the physical and chemical property of soil and increase the uptake of heavy metals by certain plants [7]. Some agricultural operations such as foliar fertilization aiming to improve application efficiency might further increase the level of heavy metals absorbed by plants [8]. What’s worse, as soil ecosystems often interface with water, the extensive use of fertilizer in soil has resulted in significant heavy metal pollution in water system. As some studies found out, chemical fertilizer is among the major sources of Zn, Ni, Cd and Pb in lake sediments, groundwater and canal water [9–11], threatening the water quality and aquatic animals. Chemical fertilizer is also the major source of nitrogen pollution, contributing 17%~25% to soil and groundwater nitrate accumulation [12]. The contaminated fruit, crops, aquatic products and drinking water could then enter tables of human beings. Previous studies in China have shown that maternal Hg and As exposure could be largely credited to rice or fish intake [13, 14], while Cd and Cu exposure most probably came from vegetable intake [15]. Those toxic substances could accumulate in bodies of pregnant women, cause maternal complications, cross the placenta, and impede fetal growth. For example, some case-control studies have indicated that women who delivered LBW babies had significantly higher Cd and Ni concentration during pregnancy [16, 17]; Nitrate ingestion from drinking water has been found to be associated with thyroid diseases and methemoglobinemia [18], which could in turn reduce birth weight [19, 20].

In rural Shanxi Province, China, our previous spatial epidemiological study has discovered that the areas with high fertilizer consumption coincided with areas with higher risk of preterm birth and LBW [21, 22]. Also in rural Shanxi Province, our case-control studies further revealed that compared to pregnant women living in villages with <50 ton annual consumption of chemical fertilizer, those living in villages with ≥100 ton annual consumption of chemical fertilizer had a 4.03 (95%CI: 1.63~9.92) times higher risk of tLBW [23], 2.16 (95%CI: 1.05~4.44) times higher risk of high birth weight [24], and 2.51(95%CI:1.23~5.12) times higher risk of preterm birth [25]. Household application of phosphorus fertilizer was also found associated with risk of preterm birth (aOR=2.54, 95%CI:1.23~5.22) [25]. All the aforementioned evidence suggests that maternal chemical fertilizer exposure could be a risk factor for adverse pregnancy outcomes.

Socio-economic disadvantage is also an important risk factor for LBW [26]. Besides, it has long been found an interaction between socio-economic factors and environmental pollution on health outcomes, namely, adverse environment has a greater impact on people of lower socio-economic status (SES). For example, a study in China found that PM_1_ and SO_2_ had a greater impact on the risk of lung cancer in lower educated men compared to their higher educated counterparts [27]. Previous studies have raised two main pathways for this health inequality of environmental exposure. First, people with lower SES are more exposed to adverse environments: They are more likely to live in areas with environmental pollution [28, 29], engage in occupations with higher environmental risks [30], and benefit less from the government's environmental policies [31]. Secondly, people with lower SES often have a higher prevalence of preexisting diseases that confer a greater risk of further diseases associated with environmental hazards [32]. In other words, they are more sensitive to pollution–related health hazards. In addition, they might have less access to medical assistance or measures to reduce the exposure risk [33].

Currently, the majority of top chemical fertilizer consumers are low and middle income developing countries [4], and the prevalence of LBW is also much higher in those countries [1]. Indeed, in China, lower educated farmers have also been observed to take an excessive application of chemical fertilizer in their household farming [29], which could potentially bring about a higher chemical fertilizer exposure in their daily life. Therefore, it’s reasonable to hypothesize that women of lower SES might be more susceptible to the adverse effects of chemical fertilizer exposure. Based on data from Shanxi Province, China, the present study thus aimed to test the hypothesis, i.e., investigate the interactive effects of maternal SES and exposure to chemical fertilizer during pregnancy on risk of term LBW (tLBW). In this way, we tried to provide a better understanding of effect mechanism of this agricultural substance on population health and identify SES inequalities in tLBW related to the exposure to chemical fertilizer, thus contributing to the primary prevention and intervention for vulnerable groups.

## Methods

### Study design and population

A population-based case–control study using the mother-infant pair as analytical unit was conducted in Pingding County, Shanxi province, northern China. Homing over 345,800 residents in 2019, Pingding County had 313 villages and a total area of 139,093 hectares among which 60% was agricultural land. Birth records were extracted from the Perinatal Health Care Surveillance System, a long-term birth surveillance system established since 1993 [34]. The System monitored all women living in the Pingding County, Shanxi Province for ≥ 1 year and their fetuses/infants from the onset of pregnancy to day 42 after delivery. Altogether, there were 18,749 births from Oct, 2007 to Sep, 2012, among whom 546 were singleton tLBW without any known birth defects and having birthweight < 2,500 g and gestational age ≥ 37 weeks. The birth prevalence of tLBW was thus 29.1 per 1,000 births. From the 546 tLBW, we randomly selected 179 as cases in our present study. There were 17,345 singleton live births with ≥ 37 gestational weeks, without any known birth defects and having birth weights ≥ 2,500 g and < 4,000 g, among whom we randomly selected 204 to represent the population we targeted [35]. The participants of the present study spanned across 120 villages.

The study protocol was reviewed and approved by the Institutional Review Board of Peking University Health Sciences Center, and written informed consent was obtained from all subjects before completing the questionnaire.

### Data collection

The data source in our study consisted of three parts. The first part was the socio-demographic characteristics of women and their husbands, perinatal dietary intake, lifestyles (including smoking and drinking), residence address, and living environment (including indoor air pollution and living distance from highways), which was collected by trained health workers via a face-to-face interview using structured questionnaire within 42 days after delivery (most within 7 days). This process of data collection was part of the Perinatal Health Care Surveillance System and thus the information was directly extracted from the System. The second part of the data was the agricultural environmental exposure data at household level, including household chemical fertilizer application, household pesticide application and personal contact with pesticides. The data were collected by trained healthcare workers after selection of cases and controls in 2013. The third part was the economic and environmental data at village level, including annual amount of chemical fertilizer use per acre and annual income per capita for all 120 villages. This information was extracted from the Annals of National Economics Statistics of Pingding County in 2010 published by the Pingding Bureau of Statistics, also after selection of cases and controls. A recent article also using the data has been published elsewhere [22].

### Maternal exposure to chemical fertilizer

Maternal exposure to chemical fertilizer was measured at household level as well as village level. At household level, exposure to chemical fertilizer was dichotomous where “none exposure to chemical fertilizer” was defined as “zero use of chemical fertilizer in farming by the women’s household”, while “exposure to fertilizer” was defined as “any other than zero use of chemical fertilizer in farming by the women’s household”. At village level, the total amount of chemical fertilizer consumed (tons) per acre in 2010 of all included villages were divided into two groups using the median as the cut-off point. Therefore, “high exposure to chemical fertilizer” at village level was defined as “use of chemical fertilizer equal to or higher than the median among all villages”, while “low exposure to chemical fertilizer” was defined as “use of chemical fertilizer lower than the median”.

### Maternal socio-economic status

Aggregating four dimensions of the household socio-economic characteristics, i.e., paternal education level, maternal education level, maternal employment status, and their household annual income, an SES index was constructed through principal component analysis according to Vyas and Kumaranayake 2006 [36]. In principal component analysis, data should be either continuous ones or binary ones [36]. Therefore, data in categorical forms, including paternal education level, maternal education level and maternal employment status, were coded into binary forms while household annual income was a continuous variable and was put into model directly. A correlation matrix was constructed to ensure that all dimensions had equal weights. The Bartlett’s test of sphericity was statistically significant (*P* < 0.001), indicating it suitable to use principal component analysis here. The first four components explained 100% of the total variations of the four dimensions of SES in the original data set. Therefore, the SES index was constructed by summing the four components with the proportion of total variations being accounted by each component as weights. The SES index was then divided into two categories using the median as the cut-off point.

### Covariates

Demographic and clinical variables, namely, maternal age, infant sex, parity, maternal body mass index (BMI) before pregnancy were included in analysis. The BMI was calculated through weight/height^2^ and was divided into three groups based on body characteristics of Chinese population [37]: < 18.5 kg/m^2^ as “underweight”, ≥ 18.5 kg/m^2^ and < 24 kg/m^2^ as “normal weight” and ≥ 24 kg/m^2^ as “overweight or obesity”.

Aside from maternal SES index, village-level economic status (namely, village income per capita), was also included in analysis. Income per capita of all villages was dichotomized using the median 6,527 RMB/year (equivalent of 955 US Dollars in 2010) as the cut-off point.

Exposure to environmental pollutants during pregnancy may as well come from other sources besides chemical fertilizer. As pesticides are commonly used together with chemical fertilizer and were also observed to be associated with tLBW in some literature [38], we quantified the pesticide exposure with two questions: “how many pesticides or herbicides were used in your household farms during the year of pregnancy” and “Did you have personal contact with pesticides or herbicides during pregnancy?”. We also tried to include variables that characterize air pollution based on previous literature [39, 40]: living distance from highways was analyzed to measure outdoor traffic air and noise pollution; indoor air pollution from coal combustion (IAPCC) [41] to measure indoor air pollution caused by coal fuels; and active and passive smoking to measure tobacco exposure.

We also analyzed maternal dietary factors during pregnancy including intake of meat (including land-based animal foods such as beef, lamb, pork, chicken, and ducks and aquatic-based animal foods like seafood, freshwater fish, and lobster), eggs or milk, fresh vegetables, and fresh fruit. Participants were asked about the frequencies of eating the food and the answers were grouped into “ ≤ 1 times per week”, “2 ~ 3 times per week”, “4 ~ 5 times per week”, and “6 ~ 7 times per week”.

### Statistical analysis

Univariate analysis was performed by comparing tLBW cases and controls in terms of chemical fertilizer exposure and covariates through chi-square test (for categorical variables), Kruskal–Wallis test (for continuous variables) or Fisher exact test (for some groups with small sample size). Multivariable logistic regression was then performed to estimate the association of chemical fertilizer exposure and SES with the risks of tLBW, adjusting for variables that were statistically significant (*P* < 0.05) in univariate analysis. The logistic regression equation was as follows:

$$Logit\left(odds\right)={\alpha +\beta }_{1}VF+{\beta }_{2}IF+{\beta }_{3}SES+\Upsilon C+\varepsilon$$

where $odds=P({Y}_{i}=1)/(1-P\left({Y}_{i}=1\right))$, ${Y}_{i}=1$ meant the pregnancy outcome was a tLBW case; $\alpha$ was the intercept of the regression; $VF$ was the chemical fertilizer exposure at village level; $IF$ was the chemical fertilizer exposure at household level; $SES$ was the socio-economic index; C was the adjusted covariates; ${\beta }_{1}$, ${\beta }_{2}$, ${\beta }_{3}$, and $\Upsilon$ were the coefficients of $VF, IF, SES$ and C; $\varepsilon$ was the error term. The adjusted ORs were then calculated as the exponential of ${\beta }_{1}$, ${\beta }_{2}$, and ${\beta }_{3}$.

To evaluate the additive interaction between maternal SES and village-level chemical fertilizer exposure in logistic regression [42], the two factors were coded into four groups: (A) those with high maternal SES and low exposure to village-level chemical fertilizer consumption; (B) those with high maternal SES but high exposure to village-level chemical fertilizer consumption; (C) those with low maternal SES but low exposure to village-level chemical fertilizer consumption; and (D) those with low maternal SES and high exposure to village-level chemical fertilizer consumption. The above groups were entered simultaneously into the multivariate logistic regression models with the (A) group as the reference group to predict the odds of tLBW, also adjusting for statistically significant variables (*P* < 0.05) in univariate analysis. The logistic regression equation was as follows:

$$Logit\left(odds\right)={\alpha +\beta }_{1}{G}_{B}+{\beta }_{2}{G}_{C}+{\beta }_{3}{G}_{D}+\Upsilon C+\varepsilon$$

where $odds=P({Y}_{i}=1)/(1-P\left({Y}_{i}=1\right))$, ${Y}_{i}=1$ meant the pregnancy outcome was a tLBW case; $\alpha$ was the intercept of the regression; ${G}_{B}$, ${G}_{C}$ and ${G}_{D}$ were used to represent the aforementioned B, C, and D group of different combinations of chemical fertilizer exposure and SES. To be specific:

${G}_{B}=1$, if high maternal SES and high exposure to village-level chemical fertilizer consumption; ${G}_{B}=0$, if otherwise.

${G}_{C}=1$, if low maternal SES and low exposure to village-level chemical fertilizer consumption; ${G}_{C}=0$, if otherwise.

${G}_{D}=1$, if low maternal SES and high exposure to village-level chemical fertilizer consumption; ${G}_{D}=0$, if otherwise.

C was adjusted covariates; ${\beta }_{1}$, ${\beta }_{2}$, ${\beta }_{3}$, and $\Upsilon$ were the coefficients of ${G}_{B},{G}_{C}, {G}_{D}$, and C; $\varepsilon$ was the error term. The adjusted ORs were calculated as the exponential of ${\beta }_{1}$, ${\beta }_{2}$, and ${\beta }_{3}$. The joint effects of SES and chemical fertilizer exposure could be reflected by the adjusted OR of ${G}_{D}$, i.e., $\mathrm{exp}({\beta }_{3})$.

In addition, we calculated the relative excess risk due to interaction (RERI) to evaluate whether the effect of SES and chemical fertilizer exposure together would exceed the effect of each considered separately. That is, we assessed whether the odds of tLBW for the group (D) were significantly greater than the sum of the odds for group (A) and (B) by calculating:

$$RERI=\left({OR}_{11}-1\right)-({OR}_{10}-1)-({OR}_{01}-1)$$

where ${OR}_{ij}$ denoted the odds ratio in exposure category i,j; $i=1$ represented low SES and $j=1$ represented high exposure to village-level chemical fertilizer consumption. Thus the ${OR}_{11}$, ${OR}_{10}$ and ${OR}_{01}$ were the odds ratio of the above B, C, and D group, namely, the exponential of ${\beta }_{1}$,${\beta }_{2}$, and${\beta }_{3}$. The Inverse Probability of Treatment Weighting Approach was adopted here to estimate RERI and obtain its 95%CI and *P*-value, as this method allows for the estimation of RERI in case–control data without having to rely on modeling assumptions for the outcome conditional on the exposures and covariates [43]. For test of *P*-value, the null hypothesis was RERI = 0. Therefore, *P* < 0.05 would suggest that RERI was significantly greater than 0. RERI > 0 would indicate an additive interaction and RERI > 1 indicate a strong interaction. Details of explanation of additive interaction and construction of RERI were described elsewhere [43].

The interaction between SES and exposure to chemical fertilizer at household level was similarly assessed.

### Sensitivity analysis

Several sensitivity analyses were carried out to test the robustness of our study. As pesticides could play crucial role in risk of tLBW and usually be applied with chemical fertilizer, household pesticide application was adjusted in multivariate models regardless its *P* value in univariate analysis. Besides, we took a lesser restrictive threshold (*P* < 0.2 rather than < 0.05 in univariate analysis) of choosing adjusted covariates in multivariate models, considering the limited sample size of our study. We also used different cut-off points of village chemical fertilizer (a 2/3 cut-off point: top 66.7% of all samples as the “high exposure” group and the rest as the “low exposure” group; and a 3/4 cut-off point: top 75% of all samples as the “high exposure” group and the rest as the “low exposure” group) to see the robustness and efficiency of the main models.

All analyses were performed using SAS 9.4.

## Results

### Basic characteristics of study participants

Table 1 displays the socio-demographic characteristics and environmental and dietary exposure of tLBW cases and controls. Our study consisted mainly of women under 28 years old (57.18%), with middle school education or lower (83.56%), being unemployed or farmers (84.33%), coming from families with median annual income of 20,000 RMB, and whose husbands were of middle school education or lower (81.20%). Maternal active smoking was rare among participants (only 2 participants had active smoking during pregnancy), yet passive smoking was more common (64.23% had passive smoking). Household pesticide application as well as maternal personal contact with pesticides during pregnancy also took a minimal share of all participants (2.61% and 1.57%, respectively). Univariate analysis showed that mothers of tLBW were more likely to be of low SES index, to eat meat ≤ 1 times/week, to eat eggs or milk < 4 times/week, to be exposed to household chemical fertilizer use, and to be highly exposed to village-level chemical fertilizer consumption (*P* < 0.05). Therefore, meat intake and eggs or milk intake were adjusted for in later multivariate analysis.

**Table 1:** Socio-demographic characteristics and environmental and dietary exposures of term low birth weight cases and controls

**Variable**	**tLBW Case**	**Control**	${{\varvec{\chi}}}^{2}$ ^a^	**P**
	**N(%)**	**N(%)**		
**Socio-demographic characteristics**				
Maternal age				
< 28	97(54.19)	122(59.80)		
≥ 28	82(45.81)	82(40.20)	1.237	0.268
Infant sex				
Female	95(53.07)	98(48.04)		
Male	84(46.93)	106(51.96)	0.996	0.326
Parity				
1	83(46.37)	103(50.49)		
≥ 2	96(53.63)	101(49.51)	0.648	0.421
Maternal BMI (kg/m^2^)				
< 18.5	8(4.47)	10(4.90)		
18.5 ~ 24	111(62.01)	131(64.22)		
≥ 24	60(33.52)	63(30.88)	0.318	0.853
Village income per capita (RMB/year)				
< 6527^b^	92(51.40)	98(48.04)		
≥ 6527	87(48.60)	106(51.96)	0.430	0.512
Individual SES index				
High	74 (41.34)	117(57.35)		
Low	105(58.66)	87(42.35)	7.778	0.002
Maternal occupation				
Others	41(22.91)	59(28.92)		
Famers or unemployed	138(77.09)	145(71.08)	1.789	0.181
Maternal education				
High schools or higher	27(15.08)	36(17.65)		
Middle schools or lower	152(84.92)	168(82.35)	0.456	0.500
Paternal education				
High schools or higher	37(20.67)	35(17.16)		
Middle schools or lower	142(79.33)	169(82.64)	0.771	0.380
Household income (Thousand RMB/year)				
Medium (Min ~ Max)	15 (2 ~ 100)	20 (2 ~ 150)	6.334	0.012
**Environmental exposure and lifestyles during pregnancy**				
Household chemical fertilizer application				
None	90(50.28)	126(61.76)		
Yes	89(49.72)	78(38.24)	5.115	0.024
Village chemical fertilizer application				
Low	72(43.11)	106(56.68)		
High	95(56.89)	81(43.32)	6.499	0.011
Household pesticide application				
No	173(96.65)	200(98.04)		
Yes	6(3.35)	4(1.96)		0.525^c^
Maternal personal contact with pesticides				
No	157(99.37)	195(97.50)		
Yes	1(0.63)	5(2.50)		0.234^c^
Living distance from highway (m)				
≤ 1000	45(25.41)	64(31.37)		
> 1000	134(74.86)	140(68.63)	1.819	0.177
IAPCC				
0	80(44.69)	111(54.41)		
≥ 1	99(55.31)	93(45.59)	3.602	0.058
Maternal active smoking				
No	177(100.00)	197(98.99)		
Yes	0(0.00)	2(1.01)		0.500^c^
Maternal passive smoking				
No	61(34.08)	76(37.52)		
Yes	118(65.92)	128(62.75)	0.419	0.518
**Dietary exposure during pregnancy**				
Meat intake (times/week)				
≤ 1	125(69.83)	120(59.11)		
2~7	54(30.17)	83(40.89)	4.752	0.029
Eggs or milk intake (times/week)				
< 4	94(52.51)	69(33.82)		
4~7	85(47.49)	135(66.18)	13.624	< 0.001
Fresh vegetables intake (times/week)				
≤ 5	114(63.69)	126(61.76)		
6~7	65(36.31)	78(38.24)	0.151	0.698
Fresh fruit intake (times/week)				
< 4	56(31.46)	54(26.47)		
4~7	122(68.54)	150(73.53)	1.154	0.283

^a^Wald χ.^2^ test for categorical variables and Kruskal–Wallis test for continuous variables

^b^the median income per capita of the 120 villages

^c^Fisher exact test

*tLBW* term low birth weight, *SES index* socio-economic status index, *IAPCC* indoor air pollution from coal combustion

### The association between chemical fertilizer exposure, SES and risk of tLBW

Table 2 displays the association of maternal exposure to chemical fertilizer and SES with the risk of tLBW. After adjusting for meat intake and eggs or milk intake, high exposure to village-level chemical fertilizer consumption was associated with increased risk of tLBW (aOR: 1.63; 95%CI: 1.06 ~ 2.51); maternal low SES was also associated with increased risk of tLBW (aOR: 1.59; 95%CI: 1.03 ~ 2.45).

**Table 2:** Association between maternal exposure to chemical fertilizer and socio-economic status with the risk of term low birth weight

Exposure factors	Unadjusted OR (95%CI)	Adjusted OR (95%CI)^a^
High exposure to household chemical fertilizer use	1.60(1.06 ~ 2.40)*	1.36(0.87 ~ 2.12)
High exposure to village-level chemical fertilizer consumption	1.72(1.15 ~ 2.57)**	1.63(1.06 ~ 2.51)*
Low socio-economic status	1.91(1.27 ~ 2.87) **	1.59(1.03 ~ 2.45)*

^a^adjusted for meat intake, eggs or milk intake

^*^P < 0.05,**P < 0.01

### Interactions between SES and chemical fertilizer exposure

Table 3 presents results of interactions between maternal SES and exposure to chemical fertilizer on the risk of tLBW. Compared to high maternal SES and low exposure to village-level chemical fertilizer consumption, the combination of low maternal SES and high exposure to village-level chemical fertilizer consumption was associated with increased risk of tLBW (aOR = 2.62, 95%CI: 1.44 ~ 4.77) after adjusting for meat intake, eggs or milk intake and exposure to household chemical fertilizer use. Similarly, compared to high maternal SES and none exposure to household chemical fertilizer use, the combination of low SES and exposure to household chemical fertilizer use also had elevated risk of tLBW (aOR = 2.18, 95%CI: 1.24 ~ 3.83) after adjusting for meat intake, eggs or milk intake and exposure to village-level chemical fertilizer consumption. The RERI for village-level chemical fertilizer exposure and SES equaled to 1.79 (95%CI: 1.11 ~ 2.47, *P* < 0.001), a strong indication that the estimated joint effect of high exposure to village-level chemical fertilizer and low SES together on tLBW was greater than the sum of the estimated effect of high exposure to village-level chemical fertilizer alone and low SES alone. Similarly, the RERI for household chemical fertilizer exposure and SES equaled to 0.77 (95%CI: 0.14 ~ 1.40, *P* < 0.05), meaning that there were some, though not strong, indications that the estimated joint effect of exposure to household chemical fertilizer use and low SES together was greater than the sum of the estimated effect of the two factors alone.

**Table 3:** Interactions between maternal socio-economic status and exposure to chemical fertilizer on the risk of low birth weight

SES	Exposure to chemical fertilizer	Cases	Controls	aOR(95%CI)	RERI (95%CI)	P
	**Village level** ^a^					
High	Low	40	66	1.00		
High	High	34	51	1.07(0.59 ~ 1.96)		
Low	Low	36	48	1.04(0.56 ~ 1.91)		
Low	High	69	39	2.62(1.44 ~ 4.77)	1.79(1.11 ~ 2.47)	< 0.001
	**Household level** ^b^					
High	None	48	81	1.00		
High	Yes	26	36	1.25(0.66 ~ 2.35)		
Low	None	42	45	1.48(0.84 ~ 2.62)		
Low	Yes	63	42	2.18(1.24 ~ 3.83)	0.77(0.14 ~ 1.40)	< 0.05

^a^OR were adjusted for meat intake, eggs or milk intake and exposure to household chemical fertilizer use

^b^OR were adjusted for meat intake, eggs or milk intake and exposure to village-level chemical fertilizer consumption

*SES* socio-economic status index, *RERI* relative excess risk due to interaction

### Results of sensitivity analysis

Additional adjustments for household pesticide application on the basis of the original models hardly changed the estimate yet generated a slightly larger variance (Table S1 and S2). Besides meat intake and eggs or milk intake, distance from highway and IAPCC also had *P* value < 0.2 in univariate analysis and were thus also adjusted in multivariate models. As seen in Table S3 and Table S4, there were only minor changes of estimation of association between chemical fertilizer exposure, SES and risk of tLBW, as well as RERI for interaction between village chemical fertilizer application and SES. While a larger RERI (RERI = 1.17, 95%CI: 0.41 ~ 1.94, *P* < 0.01) for interaction between household chemical fertilizer application and SES was found. Taking 2/3 as the cut-off point for village chemical fertilizer application didn’t change much to our results, while after taking 3/4 as the cut-off point, the association between SES and risk of preterm birth and the RERI for interaction between village chemical fertilizer application and SES became insignificant (RERI = 0.89, P = 0.071) (Table S5, S6, S7), probably because of a relatively larger standard error resulting from taking a more extreme value for grouping and thus limited sample size in certain groups.

## Discussion

This study sought to examine the associations of maternal SES, exposure to chemical fertilizer during pregnancy and their interactions with risk of tLBW. There were three main findings of our study. Firstly, after adjusting for potential confounders, chemical fertilizer exposure remained to be risk factors for tLBW. The findings were consistent with our previous researches also in Shanxi Province that risk of tLBW was associated with village chemical fertilizer consumption with a dose–response relationship [21, 23]. With mass agricultural production, Cu, As, Cd, Pb and other heavy metals originating from raw materials in chemical fertilizer production, as well as nitrates from nitrogen fertilizer, might easily concentrate in soil [5, 44]. They might then be washed out into lakes and underground water, pollute local crops, aquatic plants and animals and drinking water and finally enter dining tables of mothers and their offspring. A survey conducted in 2013 by local Bureau of Statistics in Shaanxi, a province adjacent to our setting Shanxi Province, found that over 56.8% farmers continued farming mainly for growing food for their own and their families to eat, with grain taking the largest share of their plantation land (50.2%), with vegetables and fruits taking 17.7% and 7.4% respectively [45]. Water could be another path that toxic substance from chemical fertilizer application entered into body of pregnant women. In rural areas of Shanxi, a study in 2008 has shown that the majority of residents took ground water as their drinking source and the main type of water supply was non-centralized water supply (i.e., households directly took water from rivers, lakes or wells without any treatment). Yet the qualification rate of water from non-centralized water supply was only about 57% and arsenic, nitrate nitrogen, and sulfate were among those exceeding the standard limit [46]. Considering that the application of chemical fertilizer is a non-negligible factor contributing to a high level of arsenic and a variety of heavy metals as well as nitrates in the water body and soil [5, 44, 47], and as a consequence, in grain, vegetables and aquatic food [6–10, 15], we speculated that the main path of chemical fertilizer exposure for pregnant women could be water and food contaminated by heavy metals and nitrates. A large body of research has alarmed that prenatal heavy metal could lead to intrauterine growth retardation or LBW [16, 17] by altering DNA methylation, lowering levels of insulin-like growth factors, and increasing fetal corticosterone concentrations [47–49]. Moreover, nitrites and nitrates in drinking water were suggested to be related to reduced birth weight [50], possibly through causing methemoglobinemia of pregnant women or acting as an endocrine disrupter to gonadal steroidogenesis and thyroid function in mothers and their babies, all of which might lead to LBW [19, 20, 51]. Given that China is among the largest consumers of chemical fertilizer, the adverse pregnancy outcomes of maternal exposure to those agricultural pollutants deserve public attention.

Secondly, lower SES was associated with higher risk of tLBW. SES is one of the major causes of health disparities as it underlies three major determinants of health: health care, environmental exposure, and health behavior [52]. Women in low SES, including those with low educational attainment, with low household income, being unemployed or in badly-paid occupation, were often affected by undernutrition or unbalanced diet, inadequate access to health services, bad lifestyles, passive smoking, and poor living environment [53–55], all of which might lead to maternal complications and thus LBW [56–59]. Mitigating social inequalities in health should be a concern of public policy.

Thirdly, the interactive analyses in the present study indicated that high exposure to chemical fertilizer and SES disadvantage would reinforce the risk of LBW. Similar evidence was reflected in air pollution analyses that showed effect modification by some indicators of SES for particular matter on the risk of LBW, i.e., risks of LBW associated with maternal exposure to particular matter were higher in lower SES group [59]; It was also observed that negative effects of prenatal pesticide exposure on offspring development could be enhanced by low social class [60]. In our case, several pathways could be used to explain this interaction. One likely explanation could be differential effects of exposure to chemical fertilizer by different SES groups. As indicated by some research in China, farmers of lower educational level tended to overuse chemical fertilizer [29], and were thus more exposed to chemical fertilizer. Furthermore, even if they are exposed to the same dose of chemical fertilizer at household and village levels, women from higher SES groups might be more concerned about the quality of food and water, and hence the possibility decreased of poisonous pollutants from the chemical fertilizer going into their diet. Some studies did find higher internal concentrations of heavy metals in lower SES population [61]. Another explanation could be socio-economic disparities in adverse birth outcomes [26]. As mentioned above, women with lower SES have already had higher risk of preexisting complications and often receive inferior medical treatment for those complications. As a result, they are more susceptible to environmental hazards. Yet mechanisms of relationship between environmental pollution and adverse pregnancy outcomes are complex and remain to be explored. Our study provided initial evidence for SES’s potential role in this relationship. More epidemiological studies of massive population and biological marker analyses should be combined to further explore the mechanism of association between prenatal environmental exposure, SES and adverse pregnancy outcomes.

There are some strengths of our study. First, by aggregating over different dimensions of SES and constructing SES index with principal component analysis, we minimized potential bias that might be brought about by single indicator, thus improving the test power and the reliability. Second, controls were randomly sampled from the same large surveillance database as cases, and thus could represent the population from which the case group was derived and therefore minimize selection bias. In addition, the data of village chemical fertilizer consumption were extracted from official statistics, and interviews were conducted face-to-face by well-trained healthcare workers to ensure data accuracy.

There are also several limitations that should be acknowledged. The first is the unavailability of the data of village-level pesticide consumption. Some previous literature has suggested potential effects of residential exposure to pesticide application on LBW. For example, a large scale population-based case–control study in California, USA found that first and second trimester exposure to agriculturally applied myclobutanil within 2 km of residential addresses was associated with higher risk of tLBW (OR: 1.11; 95% CI: 1.04 ~ 1.20) [38]; Studies in Brazil and Spain also found higher prevalence of LBW in regions with higher pesticide consumption [62, 63]. If village-level pesticide exposure did raise the odds of tLBW, and if there existed a positive association between pesticide application and chemical fertilizer exposure, failure to adjust the village-level pesticide exposure would overestimate the magnitude of our estimated association between chemical fertilizer and tLBW. Besides, in constructing maternal SES index, occupation of husbands, who were mostly the bread-winners of families in the study area, should have been included. Yet this variable was not available in our data. Secondly, the present study used the village chemical fertilizer consumption in 2010 to represent village-level chemical fertilizer exposure for births between October 2007 and September 2012, which means that the measured exposure might not accurately overlap with time of pregnancies. Nevertheless, the effects of chemical fertilizer exposure are very likely to be a cumulated one, and areas of the farm land, plantation and agricultural operation practice of chemical fertilizer were presumably stable during the 5-year period. Therefore, our method of measuring village-level chemical fertilizer exposure would not bring much bias to the analysis. Thirdly, information on household chemical fertilizer use, diet and lifestyles was collected after pregnancy, which might generate recall bias. In particular, the information about household chemical fertilizer use during pregnancy was collected relatively long after delivery (at maximum 5 years), and we could only assume that the agricultural practice would not change much and recalling whether chemical fertilizer was used in recent years would not be very difficult for farmers. And we have tried to minimize the recall bias by conducting the interviews of maternal socio-demographic information, diet and lifestyles within 42 days after delivery (most within 7 days). Fourthly, though we have tried to capture the chemical fertilizer exposure by using both household and village chemical fertilizer consumption data, there could be also be measurement error, as we didn’t distinguish different types of chemical fertilizer, nor could we get a comprehensive and precise measurement of how much toxic substance from chemical fertilizer entered into the body of pregnant women. The measurement error might potentially generate dilution bias in our regression models and cause overestimation or underestimation of the integrative effects. Future studies can consider improving tools of exposure measurement. For example, investigating the application of different types of chemical fertilizer can help explore their individual and combined effects; Investigating whether women used any protection equipment during farm work can help get a better idea of the degree of exposure to agricultural chemical pollutants.

## Conclusions

In conclusion, the present study suggested that maternal exposure to chemical fertilizer, low SES and their interaction might be associated with increased risk of tLBW. Our study calls for attention from public and government on adverse effects of agricultural pollutants on pregnancy outcomes and on health of socio-economic disadvantaged groups. Future research can assess the interaction between maternal exposure to agricultural pollutants, dietary factors and SES focusing on the epigenetic modification, explore its eventual effect on adverse pregnancy outcomes, and provide more information on the reduction and prevention of the occurrence of adverse pregnancy outcomes.

## Supplementary Information

**Additional file 1: Table S1.** Comparison of original model and the model adjusting additionally for pesticide application in examining association between chemical fertilizer exposure, socio-economic status and risk of tLBW. **Table S2.** Comparison of the original model and the model additionally adjusting for pesticide application in examining the interactive effects of chemical fertilizer exposure and SES on risk of tLBW. **Table S3**. Comparison of the original model and the model adjusting for variables with a p-value threshold at 0.2 in univariate analysis when examining the association between chemical fertilizer exposure, socio-economic status and risk of tLBW. **Table S4.** Comparison of the original model and the model adjusting for variables with a p-value threshold at 0.2 in univariate analysis when examining the interactive effects of chemical fertilizer exposure and SES on risk of tLBW. **Table S5.** Distribution of chemical fertilizer exposure when taking different cut-off points. **Table S6.** Comparison of results of models in examining the interactive effects of chemical fertilizer exposure and SES on risk of tLBW when taking different cut-off points of village chemical fertilizer application a. **Table S7**. Interactions between maternal socio-economic status and exposure to chemical fertilizer on the risk of low birth weight when taking 2/3 as cut-off point for village chemical fertilizer application. **Table S8.** Interactions between maternal socio-economic status and exposure to chemical fertilizer on the risk of low birth weight when taking 3/4 as cut-off point for village chemical fertilizer application.

## Data Availability

The data used to support the findings of this study are available from the corresponding author upon reasonable request.

## Abbreviations

tLBW: Term low birth weight
SES: Socio-economic status
RERI: Relative excess risk due to interaction
BMI: Body mass index
