# Supplementary material for: Interactive effects of maternal exposure to chemical fertilizer and socio-economic status on the risk of low birth weight
Source: BMC Public Health. 2022 Jun 16;22:1206. doi: 10.1186/s12889-022-13604-z (PMC9204990; doi:10.1186/s12889-022-13604-z)
Supplement: Supplementary file 1 — Additional file 1: Table S1. Comparison of original model and the model adjusting additionally for pesticide application in examining association between chemical fertilizer exposure, socio-economic status and risk of tLBW. Table S2. Comparison of the original model and the model additionally adjusting for pesticide application in examining the interactive effects of chemical fertilizer exposure and SES on risk of tLBW. Table S3. Comparison of the original model and the model adjusting for variables with a p-value threshold at 0.2 in univariate analysis when examining the association between chemical fertilizer exposure, socio-economic status and risk of tLBW. Table S4. Comparison of the original model and the model adjusting for variables with a p-value threshold at 0.2 in univariate analysis when examining the interactive effects of chemical fertilizer exposure and SES on risk of tLBW. Table S5. Distribution of chemical fertilizer exposure when taking different cut-off points. Table S6. Comparison of results of models in examining the interactive effects of chemical fertilizer exposure and SES on risk of tLBW when taking different cut-off points of village chemical fertilizer application a. Table S7. Interactions between maternal socio-economic status and exposure to chemical fertilizer on the risk of low birth weight when taking 2/3 as cut-off point for village chemical fertilizer application. Table S8. Interactions between maternal socio-economic status and exposure to chemical fertilizer on the risk of low birth weight when taking 3/4 as cut-off point for village chemical fertilizer application. [file 12889_2022_13604_MOESM1_ESM.docx]

**SUPPLEMENTARY MATERIAL**

**Interactive effects of maternal exposure to chemical fertilizer and socio-economic status on the risk of low birth weight**

Shiqi Lin^1,*^, Jiajia Li^1,*^, Jilei Wu^1^, Fan Yang^1^, Lijun Pei^1,#^, Xuejun Shang^2,#^

^1^Institute of Population Research/China Center on Population Health and Development, Peking University, Beijing, 100871, China;

^2^ Department of Andrology, Jinling Hospital, School of Medicine, Nanjing University, Jiangsu, 210002, China

**Table of contents**

[Table S1 Comparison of original model and the model adjusting additionally for pesticide application in examining association between chemical fertilizer exposure, socio-economic status and risk of tLBW 2](#_Toc106263653)

[Table S2 Comparison of the original model and the model additionally adjusting for pesticide application in examining the interactive effects of chemical fertilizer exposure and SES on risk of tLBW 2](#_Toc106263654)

[Table S3 Comparison of the original model and the model adjusting for variables with a p-value threshold at 0.2 in univariate analysis when examining the association between chemical fertilizer exposure, socio-economic status and risk of tLBW 3](#_Toc106263655)

[Table S4 Comparison of the original model and the model adjusting for variables with a p-value threshold at 0.2 in univariate analysis when examining the interactive effects of chemical fertilizer exposure and SES on risk of tLBW 3](#_Toc106263656)

[Table S5 Distribution of chemical fertilizer exposure when taking different cut-off points. 4](#_Toc106263657)

[Table S6 Comparison of results of models in examining the interactive effects of chemical fertilizer exposure and SES on risk of tLBW when taking different cut-off points of village chemical fertilizer application a 4](#_Toc106263658)

[Table S7 Interactions between maternal socio-economic status and exposure to chemical fertilizer on the risk of low birth weight when taking 2/3 as cut-off point for village chemical fertilizer application 4](#_Toc106263659)

[Table S8 Interactions between maternal socio-economic status and exposure to chemical fertilizer on the risk of low birth weight when taking 3/4 as cut-off point for village chemical fertilizer application 5](#_Toc106263660)

# Table S1 Comparison of original model and the model adjusting additionally for pesticide application in examining association between chemical fertilizer exposure, socio-economic status and risk of tLBW

| **Exposure factors** | **OR(95%CI)** | |
| --- | --- | --- |
|  | **Original model ^a^** | **Model 2^b^** |
| High exposure to household chemical fertilizer use | 1.36(0.87~2.12) | 1.34 (0.85~2.10) |
| High exposure to village-level chemical fertilizer consumption | 1.63(1.06~2.51)* | 1.63(1.06~2.51)* |
| Low socio-economic status | 1.59(1.03~2.45)* | 1.59(1.03~2.46)* |

^a^ adjusted for meat intake, eggs or milk intake;

^b^ adjusted for meat intake, eggs or milk intake and household pesticide application;

* P<0.05,**P<0.01

# Table S2 Comparison of the original model and the model additionally adjusting for pesticide application in examining the interactive effects of chemical fertilizer exposure and SES on risk of tLBW

| **SES** | **Exposure to chemical fertilizer** | **Original model** | | **Model 2** | | |
| --- | --- | --- | --- | --- | --- | --- |
|  |  | **aOR(95%CI)** | **RERI (95%CI)** | **aOR(95%CI)** | **RERI (95%CI)** | |
|  | **Village level** ^a^ |  |  |  | |  |
| High | Low | 1.00 |  | 1.00 | |  |
| High | High | 1.07(0.59~1.96) |  | 1.06(0.58~1.95) | |  |
| Low | Low | 1.04(0.56~1.91) |  | 1.04(0.56~1.90) | |  |
| Low | High | 2.62(1.44~4.77) | 1.79(1.11~2.47) | 2.63(1.45~4.78) | | 1.98(1.26~2.70) |
|  | **Household level** ^b^ |  |  |  | |  |
| High | None | 1.00 |  | 1.00 | |  |
| High | Yes | 1.25(0.66~2.35) |  | 1.22(0.64~2.33) | |  |
| Low | None | 1.48(0.84~2.62) |  | 1.48(0.84~2.62) | |  |
| Low | Yes | 2.18(1.24~3.83) | 0.77(0.14~1.40) | 2.15(1.22~3.79) | | 0.76(0.13~1.39) |

^a^ In original model, OR were adjusted for meat intake, eggs or milk intake and exposure to household chemical fertilizer use, while in model 2, pesticide application was additionally adjusted.

^b^ In original model, OR were adjusted for meat intake, eggs or milk intake and exposure to village-level chemical fertilizer consumption, while in model 2, pesticide application was additionally adjusted.

SES: socio-economic status index; RERI: relative excess risk due to interaction;

# Table S3 Comparison of the original model and the model adjusting for variables with a p-value threshold at 0.2 in univariate analysis when examining the association between chemical fertilizer exposure, socio-economic status and risk of tLBW

| **Exposure factors** | **OR(95%CI)** | |
| --- | --- | --- |
|  | **Original model ^a^** | **Model 2^b^** |
| High exposure to household chemical fertilizer use | 1.36(0.87~2.12) | 1.29 (0.81~2.06) |
| High exposure to village-level chemical fertilizer consumption | 1.63(1.06~2.51)* | 1.61(1.04~2.48)* |
| Low socio-economic status | 1.59(1.03~2.45)* | 1.55(0.99~2.44) |

^a^ adjusted for meat intake, eggs or milk intake;

^b^ adjusted for meat intake, eggs or milk intake, distance from highway and IAPCC;

* P<0.05,**P<0.01

# Table S4 Comparison of the original model and the model adjusting for variables with a p-value threshold at 0.2 in univariate analysis when examining the interactive effects of chemical fertilizer exposure and SES on risk of tLBW

| **SES** | **Exposure to chemical fertilizer** | **Original model** | | **Model 2** | | |
| --- | --- | --- | --- | --- | --- | --- |
|  |  | **aOR(95%CI)** | **RERI (95%CI)** | **aOR(95%CI)** | **RERI (95%CI)** | |
|  | **Village level** ^a^ |  |  |  | |  |
| High | Low | 1.00 |  | 1.00 | |  |
| High | High | 1.07(0.59~1.96) |  | 1.05(0.57~1.93) | |  |
| Low | Low | 1.04(0.56~1.91) |  | 1.02(0.55~1.88) | |  |
| Low | High | 2.62(1.44~4.77)** | 1.79(1.11~2.47)** | 2.55 (1.38~4.72)** | | 1.61(0.97~2.25)** |
|  | **Household level** ^b^ |  |  |  | |  |
| High | None | 1.00 |  | 1.00 | |  |
| High | Yes | 1.25(0.66~2.35) |  | 1.20(0.63~2.29) | |  |
| Low | None | 1.48(0.84~2.62) |  | 1.46(0.81~2.61) | |  |
| Low | Yes | 2.18(1.24~3.83)** | 0.77(0.14~1.40)* | 2.02(1.08~3.77)* | | 1.17(0.41~1.94)** |

^a^ In original model, OR were adjusted for meat intake, eggs or milk intake and exposure to household chemical fertilizer use, while in model 2, distance from highway and IAPCC were additionally adjusted.

^b^ In original model, OR were adjusted for meat intake, eggs or milk intake and exposure to village-level chemical fertilizer consumption, while in model 2, distance from highway and IAPCC were additionally adjusted.

SES: socio-economic status index; RERI: relative excess risk due to interaction;

* P<0.05,**P<0.01

# Table S5 Distribution of chemical fertilizer exposure when taking different cut-off points.

| **Village chemical fertilizer application(tons/acre)** | **tLBW Case** | **Control** | $\boldsymbol{\chi}^{\boldsymbol{2}}$ ^a^ | **P** |  |
| --- | --- | --- | --- | --- | --- |
|  | **N(%)** | **N(%)** |  |  |  |
| **Taking 2/3 as cut-off point** | | | | |  |
| <61.4 | 102(56.98) | 151(74.02) |  |  |  |
| ≥61.4 | 77(43.02) | 53(25.98) | 12.432 | <0.001 |  |
| **Taking 3/4 as cut-off point** | | | | | |
| <90 | 109(60.89) | 165(80.88) |  |  |  |
| ≥90 | 70(39.11) | 39(19.12) | 18.710 | <0.001 |  |

# Table S6 Comparison of results of models in examining the interactive effects of chemical fertilizer exposure and SES on risk of tLBW when taking different cut-off points of village chemical fertilizer application a

| **Exposure factors** | **OR(95%CI)** | |
| --- | --- | --- |
|  | **Taking 2/3 as cut-off point** | **Taking 3/4 as cut-off point** |
| High exposure to household chemical fertilizer use | 1.38(0.89~2.14) | 1.33(0.85~2.08) |
| High exposure to village-level chemical fertilizer consumption | 2.09(1.33~3.29)** | 2.71(1.66~4.43) ** |
| Low socio-economic status | 1.55(1.00~2.39)* | 1.47(0.94~2.28) |

^a^ adjusted for meat intake, eggs or milk intake;

* P<0.05,**P<0.01

# Table S7 Interactions between maternal socio-economic status and exposure to chemical fertilizer on the risk of low birth weight when taking 2/3 as cut-off point for village chemical fertilizer application

| **SES** | **Exposure to chemical fertilizer** | **Cases** | **Controls** | **aOR(95%CI)** | **RERI (95%CI)** | **P** |
| --- | --- | --- | --- | --- | --- | --- |
|  | **Village level** ^a^ |  |  |  |  |  |
| High | Low | 48 | 28 | 1.00 |  |  |
| High | High | 26 | 89 | 1.70(0.88~3.30) |  |  |
| Low | Low | 54 | 28 | 1.36(0.80~2.31) |  |  |
| Low | High | 51 | 62 | 3.42(1.83~6.38) | 1.22(0.41~2.03) | <0.05 |
|  | **Household level** ^b^ |  |  |  |  |  |
| High | None | 48 | 81 | 1.00 |  |  |
| High | Yes | 26 | 36 | 1.24(0.65~2.36) |  |  |
| Low | None | 42 | 45 | 1.42(0.80~2.52) |  |  |
| Low | Yes | 63 | 42 | 2.15(1.23~3.77) | 0.89(0.27~1.50) | <0.05 |

^a^ OR were adjusted for meat intake, eggs or milk intake and exposure to household chemical fertilizer use;

^b^ OR were adjusted for meat intake, eggs or milk intake and exposure to village-level chemical fertilizer consumption

SES: socio-economic status index; RERI: relative excess risk due to interaction;

# Table S8 Interactions between maternal socio-economic status and exposure to chemical fertilizer on the risk of low birth weight when taking 3/4 as cut-off point for village chemical fertilizer application

| **SES** | **Exposure to chemical fertilizer** | **Cases** | **Controls** | **aOR(95%CI)** | **RERI (95%CI)** | **P** |
| --- | --- | --- | --- | --- | --- | --- |
|  | **Village level** ^a^ |  |  |  |  |  |
| High | Low | 51 | 100 | 1.00 |  |  |
| High | High | 23 | 17 | 2.80(1.34~5.86) |  |  |
| Low | Low | 58 | 65 | 1.49(0.89~2.49) |  |  |
| Low | High | 47 | 22 | 3.95(2.07~7.51) | 0.89(-0.08~1.86) | 0.071 |
|  | **Household level** ^b^ |  |  |  |  |  |
| High | None | 48 | 81 | 1.00 |  |  |
| High | Yes | 26 | 36 | 1.17(0.61~2.24) |  |  |
| Low | None | 42 | 45 | 1.33(0.75~2.36) |  |  |
| Low | Yes | 63 | 42 | 1.98(1.12~3.50) | 0.77(0.19~1.36) | <0.01 |

^a^ OR were adjusted for meat intake, eggs or milk intake and exposure to household chemical fertilizer use;

^b^ OR were adjusted for meat intake, eggs or milk intake and exposure to village-level chemical fertilizer consumption

SES: socio-economic status index; RERI: relative excess risk due to interaction;
